# From Structure to Strategy: Rethinking Early Detection Paradigms in Modern Ophthalmology

**DOI:** 10.22336/rjo.2026.01

**Published:** 2026

**Authors:** Ruxandra Pîrvulescu

**Affiliations:** Editor-in-Chief

Over the past decade, advances in ocular imaging - particularly optical coherence tomography (OCT) and OCT angiography (OCTA) - have significantly enhanced our ability to detect subtle structural changes in the eye. These technologies have turned Ophthalmology into an increasingly precise, quantitative discipline. However, despite this progress, the integration of early structural biomarkers into clinical decision-making remains inconsistent.

In a wide range of ocular conditions, structural alterations significantly precede functional impairment. This paradigm is well exemplified in glaucoma, where retinal nerve fiber layer (RNFL) thinning may occur before detectable visual field loss, and in ocular toxicity related to systemic diseases, such as hydroxychloroquine-induced retinopathy, where parafoveal changes in inner retinal layers can be identified prior to symptomatic decline. Similarly, in vascular retinal diseases, microvascular alterations identified on OCTA may anticipate clinically apparent retinopathy. These findings indicate that relying mainly on functional endpoints may overlook early disease changes.

Despite substantial evidence, clinical practice still largely depends on functional testing or on structural changes that are already detectable. This raises a critical question: are we prepared to redefine disease onset based on subclinical structural findings? While earlier detection may offer a theoretical window for prompt intervention, it also introduces concerns regarding overdiagnosis, patient anxiety, and the potential for unnecessary treatment. Furthermore, variability between imaging devices, differences in segmentation algorithms, and the lack of universally accepted normative databases continue to limit the standardization of structural biomarkers.

Another important aspect is confirming these results over time. While cross-sectional studies have demonstrated the sensitivity of structural imaging, fewer studies have established reliable predictive models linking early changes to clinically meaningful outcomes. In this context, artificial intelligence may help analyze large amounts of data and detect patterns that are difficult to identify with traditional methods.

From a regional perspective, the use of advanced imaging technologies is increasing in Romania, yet their implementation in routine clinical practice remains inconsistent. Establishing standardized protocols for acquisition, analysis, and interpretation could facilitate a more uniform approach and support alignment with European training and practice standards, such as those promoted by the European Board of Ophthalmology.

In simple terms, Ophthalmology is moving from detecting established diseases to identifying early structural changes. This shift reflects both technological progress and a change in ophthalmologists’ clinical perspective. However, the next challenge will be to use this increased sensitivity to improve patient outcomes while avoiding overdiagnosis and unnecessary interventions.

Assoc. Prof. Ruxandra Pîrvulescu, MD, PhD,

Editor-in-Chief

